# RETRACTION: Wound Healing Rates in COPD Patients Undergoing Traditional Pulmonary Rehabilitation Versus Tailored Wound‐Centric Interventions

**DOI:** 10.1111/iwj.70663

**Published:** 2025-04-23

## Abstract

**RETRACTION:** HeY. , ZhuH. , XuW. , WangT. , and ChenY. , “Wound Healing Rates in COPD Patients Undergoing Traditional Pulmonary Rehabilitation Versus Tailored Wound‐Centric Interventions,” International Wound Journal 21, no. 4 (2024): e14863, 10.1111/iwj.14863.38606653 PMC11009941

The above article, published online on 12 April 2024, in Wiley Online Library (http://onlinelibrary.wiley.com/), has been retracted by agreement between the journal Editor in Chief, Professor Keith Harding; and John Wiley & Sons Ltd. Following an investigation by the publisher, all parties have concluded that this article was accepted solely on the basis of a compromised peer review process. The editors have therefore decided to retract the article. The authors agree with the retraction.

**RETRACTION:** Y. He , H. Zhu , W. Xu , T. Wang , and Y. Chen , “Wound Healing Rates in COPD Patients Undergoing Traditional Pulmonary Rehabilitation Versus Tailored Wound‐Centric Interventions,” International Wound Journal 21, no. 4 (2024): e14863, 10.1111/iwj.14863.38606653 PMC11009941

The above article, published online on 12 April 2024, in Wiley Online Library (http://onlinelibrary.wiley.com/), has been retracted by agreement between the journal Editor in Chief, Professor Keith Harding; and John Wiley & Sons Ltd. Following an investigation by the publisher, all parties have concluded that this article was accepted solely on the basis of a compromised peer review process. The editors have therefore decided to retract the article. The authors agree with the retraction.

